# The effect of opium addiction on thyroid function tests

**DOI:** 10.1186/2251-6581-13-5

**Published:** 2014-01-06

**Authors:** Mohammad Hossein Gozashti, Elham Mohammadzadeh, Kouros Divsalar, Mostafa Shokoohi

**Affiliations:** 1 Physiology research center, Kerman University of Medical Sciences, Kerman, Iran; 2 Student research committee, Kerman University of Medical Sciences, Kerman, Iran; 3 Nourosciences research center, Kerman University of Medical Sciences, Kerman, Iran; 4 Research center for modeling in health, Institute for Futures Studies in Health, Kerman University of Medical Sciences, Kerman, Iran

**Keywords:** Opium, Thyroid function test, Free T4

## Abstract

**Background:**

A number of abnormalities has been identified among drug addicted users especially heroin addicts. However, there are a few studies to assess the opium effects on thyroid hormones. the aim of the present study is to investigate the effect of opium on the thyroid function tests.

**Method:**

In this case–control, 50 male addicts, aged 20–50 years, with history of addiction to opium lasting more than two years, and 50 male non-addicts as control group were randomly selected. 10 cc blood sample was taken for measurements of TSH, total T4 and T3, free T4 and T3, and T3 resin uptake (T3RU) and 50 cc urine sample for opium testing.

**Results:**

The univariate analysis revealed that there was not a significant association between opium and serum levels of T4 and TSH, but compared with control group, a slight increase in total T3 and a decrease in T3RU were observed among addicts (P < 0.05). In multivariate analysis, opium was also found to exert a lowering effect on serum free T4 level after adjusting of age and cigarette smoking (P < 0.05).

**Conclusion:**

The findings of the present study demonstrated that opium can influence on thyroid function by increasing total T3 and decreasing T3RU and free T4 levels.

## Introduction

Increasing rate of narcotics consumption is one of the major challenges in most countries. Among narcotics, opium and heroin have high rate of consumption around the world. In Iran, according to the previous studies, the prevalence of opium consumption is very high. The people of some societies especially Iran, use the opium for medication and recreational purposes [1,2]. The effect of opium on some major diseases such as coronary artery disease (CAD) [3], diabetes mellitus (DM) [4] and psychiatric symptoms [5] has been studied. In spite of many studies about the effects of morphine on body systems, the effects of long-term consumption of opium on endocrine system have not been investigated so much and most related studies have focused on the effects of heroine which is the most major consumed narcotic in western countries [6].

Studies have shown that consumption of opium derivatives can affect the function of hypothalamic-pituitary axis [7]. Although there are some studies rejecting the effect of alcohol and heroine consumption on hypothalamic-pituitary axis [8], or on T3 and T4 levels [9], some other studies have reported different results and showed decreased rate of hypothalamic-pituitary axis function [10-12]. Pfeiffer et al. showed that endogenous opioids including β-endorphin, enkephalins and dynorphins and also opiate receptors have an important role in regulation of several neuroendocrine functions [13]. It has been shown that opioids inhibit luteinizing hormone (LH) and thyrotropin secretion in rats [14]. Opioids control neuroendocrine processes in hypothalamic level through releasing hypothalamic releasing or inhibiting factors [15].

Substance abuse affects hypothalamic-pituitary-gonadal axis and body hormonal system [16]. Morphine increases hormones such as adrenalin, noradrenaline, corticosterone and glucagon [17-19] and can reduce the activity of hypothalamic-pituitary axis [20], but in regard to alterations of T3, T4 and especially TSH levels following morphine consumption, different and sometimes controversial results have been reported [20,21]. Some researchers have reported decrease of TSH level but no alterations in T3 and T4 levels during morphine consumption [20], while some others have reported decrease of TSH, T3 and T4 levels following short-term consumption of morphine [22]. According to a study performed in Pakistan, opium consumption leads to total T3 increase and total T4 decrease [23].

Considering to the controversial reports about opium effect on hypothalamic-pituitary-thyroid axis, the present study was designed to investigate alterations of thyroid function hormones among opium addicts compared with non-addicts.

## Material & methods

A total of 50 opium addict men as case group and 50 non-addict men as control group in the age range of 20–50 years were randomly selected. Continuous consumption of opium [inhalation, using pipe (locally named “Vafoor”) or heated-stone (locally named “Sikh-sang”)] for more than two years and being opium-dependent according to DSM-IV criteria were considered as inclusion criteria in addict group. Other consumers including other substance abusers but opium (such as Heroin, crystal, and methamphetamines and so on), recreational and occasional users and multi-drug users were excluded. Dependents who had history of syphilis, hepatitis and any infectious diseases with clear clinical symptoms were also excluded. Other exclusion criteria were consuming neurological or steroid drugs, history of hypo/hyperthyroidism, thyroid surgery or enlarged thyroid.

Addict subjects were selected from individuals referred to drug quit center who had not already received any medication for drug quit and had the inclusion criteria. Control group were selected from non-addict companions of patients referred to hospitals’ clinics. An informed consent form was obtained from all participants. This study was approved by Ethics Committee of Kerman University of Medical Sciences (KUMS)(ethic code: K/90/63).

### Addiction definition

According to DSM-IV criteria, the individuals who had at least three of the following seven signs during a 12-month period are considered as opium-dependent: 1) Tolerance to opium consumption, 2) Having withdrawal signs in the case of non-consumption, 3) Need for increasing opium consumption with passing of time, 4) Persistent desire for decreasing or quitting consumption, 5) Spending significant amount of time for preparing opium or freedom from this substance effects, 6) Leaving social, professional and recreational activities, 7) Persistent opium consumption in spite of knowing the physical or psychological outcomes of opium consumption.

In order to ensure persistent opium consumption, urine samples of addicts were obtained for opiates detection diagnostic tests. In the first step, a primary screening by Rapid Situation Assessment (RSA) was performed and then TLC test including liquid- solid column chromatography followed by thin layer chromatography (Akon, Baharafshan, Tehran, Iran) were performed on positive cases of the primary screening. For more assurance of non-consumption of opiates, the control group was also underwent opiates detection tests.

### Thyroid tests

Ten mL of fresh venous blood in fasting state (at 8 am) was collected and after clotting were centrifuged for 5–10 minutes to isolate the serum. The isolated serum was kept at -20°C till performing the experiments. The concentrations of thyroid parameters consisted of serum levels of TSH, total T4 and T3, free T4 and T3 and T3RU were measured by valid techniques and using Elecsis 2010 instrument. TSH level of 0.34-4.25 mIU/L was considered as normal level. All opiates detection tests in addicts and non-addicts were performed on early morning urine samples. After explaining the necessary points to the subjects, thyroid parameters in the serum of both case and control groups were measured.

### Statistical analysis

Descriptive statistics including mean and standard deviations (SD) for continuous variable and relative and absolute frequencies for categorical variables were used. Age, cigarette smoking, date of the last consumption and duration of opium dependency were recorded in a check-list. Student’s t test and chi square test (Fisher’s exact test, if necessary) were also used. Simple and multivariate linear regressions were applied in order to adjust the confounding factors such as age and cigarette smoking. Spearmanof results: MS, MHG correlation coefficient (rho) was used for identifying the association between duration of opium consumption and each of thyroid function parameters in the addict group. Data were analyzed by SPSS15 software package. P values less than 0.05 was considered statistical significant level.

## Results

Mean age and the frequency of cigarette smoking showed significant difference between the two groups, so that, mean age of addicts was significantly higher compared with non-addicts (38.6 ± 7.7 vs. 33.8 ± 8.6, respectively, P = 0.004). In addict group, 58% and in non-addicts, 38% were cigarette smoker (P = 0.045).

Total T_3_ level was significantly higher in addicts (P = 0.017), while T3RU in this group was significantly lower than that in the non-addict group (P = 0.011). There was no significant difference between the two groups in regard to other thyroid parameters (Table 1). In regard to the prevalence of thyroid disorders, based on TSH level, no statistically significant difference was observed between the two groups (Table 1).

**Table 1:** The comparison of thyroid function tests and thyroid disorder between two groups of opium addicted and control

	**Non-opium group**	**Opium group**	**P value**
	**(n = 50)**	**(n = 50)**	
Total T3 (ng/dL)	104.5 ± 25.4^*^	92.3 ± 24.4	0.017^†^
Total T4 (μg/dL)	8.08 ± 2.1	9.3 ± 13.7	0.53^†^
Free T3 (pmol/L)	2.7 ± 0.7	2.6 ± 0.6	0.66^†^
Free T4 (ng/dL)	1.2 ± 0.9	1.23 ± 0.18	0.58^†^
T3RU (%)	29.4 ± 2.3	30.5 ± 2.03	0.01^†^
TSH (mIU/L)	1.9 ± 0.9	2.2 ± 1.6	0.24^†^
**Thyroid disorder based on TSH levels**			
TSH > 4.25 mIU/L	6 (12)^**^	1 (2)	
0.34-4.25 mIU/L (Normal level)	43 (86)	48 (96)	0.11^‡^
TSH < 0.34 mIU/L	1 (2)	1 (2)	

*Mean ± SD, **N(%).

†student t test ‡Chi square test (Fisher’s exact test).

According to the results of simple univariate linear regression, total T_3_ had a significant direct association (β = 12.16, P = 0.017) and T3UR had a significant reverse association (β = -1.13, P = 0.011) with opium consumption. Other thyroid function parameters showed no significant association with opium consumption (Table 2). After adjusting the effect of age and cigarette smoking in multivariate linear regression analysis, opium consumption was significantly associated with increased serum level of total T_3_ (β = 10.2, P = 0.049) and decreased serum level of T3RU (β = -0.91, p = 0.041) and serum level of free T_4_ (β = -0.035, p = 0.001) (Table 2).

**Table 2:** The effect of opium on thyroid function test by univariate linear regression (crude analysis) and multivariate linear regression (adjusted analysis)

	**Crud analysis**^**†**^			**Adjusted analysis**^**‡**^		
	***β***	***SEM***	***P value***	***β***	***SEM***	***P value***
Total T3	12.1	4.9	0.018	10.2	5.1	0.049
Total T4	-1.2	1.9	0.53	-1.5	2.06	0.47
TSH	-0.31	0.27	0.25	-0.23	0.28	0.41
T3RU	-1.13	0.44	0.011	-0.91	0.44	0.04
Free T4	-0.21	0.04	0.58	-0.04	0.04	0.001
Free T3	0.06	0.14	0.66	0.08	0.15	0.62

†Unadjusted linear regression test ‡Adjusted linear regression test (controlling for age and cigarette smoking).

Table 3 shows the correlation between duration of opium consumption and thyroid parameters in addicts and as it is seen, among these parameters only TSH had a borderline significant reverse correlation with duration of opium consumption (P = 0.088) and other thyroid function parameters showed no significant correlation.

**Table 3:** Correlation between each thyroid parameter and duration of opium consumption among addicted group

**Thyroid parameter**	***Correlation coefficients *** **(rho)**	**P value**
Total T3	0.22	0.13
Total T4	0.24	0.10
TSH	-0.25	0.088
T3RU	-0.22	0.14
Free T4	-0.02	0.89
Free T3	-0.05	0.73

## Discussion

The results of the present study showed that addicts have higher serum level of total T_3_ and lower T3RU and free T4 compared with non-addict subjects; even though, the prevalence of thyroid disorders in the two groups had no statistically significant difference.

While previous studies have mostly investigated the effects of heroin and methadone on thyroid hormones, in the present study, the effect of opium inhalation on thyroid hormones was investigated. It has been recognized that alcohol, cocaine, and heroin have no effect on the release of pituitary hormones and probably T3 and T4 levels. But there are some reports claiming alterations of T3 and T4 levels that are mostly due to the direct effects of these substances on thyroid gland [22,23]. In another study performed in Pakistan, opium consumption has associated with increase of total T3 and decrease of total T4 levels, but TSH and free hormones levels have not been investigated in the mentioned study [24].

In a study performed on 10 heroin-dependent men, no difference was found between this group and control group in regard to TSH, T3 and T4 serum levels [8]. But in another study by Rasheed et al. about the effect of heroin on thyroid hormones, T3 level of heroin-dependents has been slightly higher than that of the control group, while TSH and T4 levels did not show significant difference between the two groups [9] Dogar et al. have reported significant elevated T3 and decreased T4 levels in the serum of substance abusers compared to the control group [24].

In a recent study by Shahsavar et al. (2013), it was shown that in comparison with non-addicts, addicts significantly had higher T3 level. They also demonstrated that addicts group had significantly lower T3 uptake level compared to the healthy group. The results of these researchers were concordant with ours [25]. The results and findings of two studies of Zhang et al. [26] and Bhoir et al. [27] in 2009 were consistent with our findings. In the present study, opium addicts showed higher total T3 and lower T3RU and free T4 levels when compared with non-addicts, while there was no significant difference between the two groups in other free hormones levels. Since just total hormones have increased, it might be due to the alteration of Thyroid Binding Protein (TBG) level. Also, according to the reduced T3RU in the addicts and reverse relationship of T3RU with TBG, some researcher concluded that opium probably cause increased TBG [25]. In fact, T3RU is the most common measurement utilized to assess TBG [25,28]. Likewise, there is a reverse association between T3RU and TBG, and T3RU measurement is used to evaluate 1/TBG [28].

Afrasiabi et al. have reported increase of total T4 and reduction of TBG and T3RU levels in heroin dependents men, but in our study total T3 showed increase, T4 was normal and T3RU showed reduction in addict subjects [29]. In Azizi et al. study, total T4 and TBG levels showed increase [30]. Alterations in total level of thyroid hormones might be due to the co-administration of other drugs and methadone in Azizi’s study or the presence of impurities in the consumed drugs by addicts.

Opiates exert their effects on hypothalamic-pituitary axis through K-receptors located in hypothalamus and their peripheral effect on liver leads to increase in production of TBG and decrease of thyroid hormones metabolism [31]. The involved mechanism causing such alterations in liver has not been known.

The effect of opiates on hypothalamic-pituitary axis and thyroid varies based on the type of consumed opiate. For example, increased thyroid-stimulating hormone (TSH) following buprenorphine and heroin consumption has been reported [32]. In a study, no difference in TBG level has been observed between heroin- dependents and normal subjects [33]. According to another study, those who received methadone maintenance therapy showed normal level of TSH and T3 responses to thyrotropin-releasing hormone administration [34].

In addition to other problems of addiction, the effect of substance abuse on thyroid, as part of the endocrine system, is worthy of paying attention. The results of the present study show the importance of general evaluation of addicts.

Further studies with longer follow up periods and more general evaluation of endocrine system and hypothalomic-pituitary axis and TBG are recommended to recognize the effects of opium and other substances abuse. The measurement of TBG may help to attain and reach more useful findings in the future studies.

## Conclusion

According to the results of this study, we conclude that opium like to methadone and heroin can cause changes in thyroid binding protein and serum levels of total t3, T3RU and free T4.

## Competing interests

All authors declare that they have no conflict of interest.

## Author’s contributions

Designing the research: MHG, EM, Gathering the data: EM, KD; Analysing the data: MS; interpretation of results: MS, MHG; drafting the manuscript: MHG, MS, EM, KD. All authors read and approved the final manuscript.
